# Impact of the 2014 American Academy of Pediatrics recommendation and of the resulting limited financial coverage by the Italian Medicines Agency for palivizumab prophylaxis on the RSV-associated hospitalizations in preterm infants during the 2016–2017 epidemic season: a systematic review of seven Italian reports

**DOI:** 10.1186/s13052-019-0736-5

**Published:** 2019-11-09

**Authors:** Renato Cutrera, Andrea Wolfler, Simonetta Picone, Giovanni A. Rossi, Giuliana Gualberti, Rocco Merolla, Antonio Del Vecchio, Alberto Villani, Fabio Midulla, Andrea Dotta

**Affiliations:** 1 Pediatric Pulmonology and Sleep & Long-Term Ventilation Unit, Pediatric Intermediate Care Unit, Academic Pediatric Department, Pediatric Hospital “Bambino Gesù”, Rome, Italy; 2 Division of Anesthesia and Intensive Care Department of Pediatrics, Children’s Hospital V Buzzi, Milan, Italy; 3 Neonatology-NICU “Casilino” General Hospital, Rome, Italy; 4 Department of Pediatrics, Pulmonology and Allergy Units, “Giannina Gaslini” Institute, Genoa, Italy; 5 Medical Department, AbbVie Italy, Rome, Italy; 6 Department of Women’s and Children Health, “Di Venere” Hospital, Bari, Italy; 7 Pediatric and Infectious Disease Unit, Academic Pediatric Department, Pediatric Hospital “Bambino Gesù”, Rome, Italy; 8 Pediatric Department, “Sapienza” University of Rome, Rome, Italy; 9 Neonatology Unit, Academic Pediatric Department, Pediatric Hospital “Bambino Gesù”, Rome, Italy

**Keywords:** Bronchiolitis, Respiratory syncytial virus, Prophylaxis, Palivizumab, Preterm infants, pediatric intensive care units

## Abstract

**Background:**

The only pharmacologic prophylaxis against respiratory syncytial virus (RSV) infection in preterm infants is the humanized monoclonal antibody palivizumab. After the 2014 modification of the American Academy of Pediatrics (AAP) recommendations, the Italian Medicines Agency (AIFA) limited the financial coverage for palivizumab prescriptions to otherwise healthy preterm infants with < 29 weeks of gestational age (wGA) aged < 12 months at the beginning of the 2016–2017 RSV season. However, due to the effect on disease severity and hospitalizations following this limitation, shown by several Italian clinical studies, in November 2017 AIFA reinstated the financial coverage for these infants. In this systematic review, we critically summarize the data that show the importance of palivizumab prophylaxis.

**Methods:**

Data from six Italian pediatric institutes and the Italian Network of Pediatric Intensive Care Units (TIPNet) were retrieved from the literature and considered. The epidemiologic information for infants 29–36 wGA, aged < 12 months and admitted for viral-induced acute lower respiratory tract infection were retrospectively reviewed. RSV-associated hospitalizations were compared between the season with running limitation, i.e. 2016–2017, versus 2 seasons before (2014–2015 and 2015–2016) and one season after (2017–2018) the AIFA limitation.

**Results:**

During the 2016–2017 RSV epidemic season, when the AIFA limited the financial coverage of palivizumab prophylaxis based on the 2014 AAP recommendation, the study reports on a higher incidences of RSV bronchiolitis and greater respiratory function impairment. During this season, we also found an increase in hospitalizations and admissions to the Pediatric Intensive Care Units and longer hospital stays, incurring higher healthcare costs. During the 2016–2017 epidemic season, an overall increase in the number of RSV bronchiolitis cases was also observed in infants born full term, suggesting that the decreased prophylaxis in preterm infants may have caused a wider infection diffusion in groups of infants not considered to be at risk.

**Conclusions:**

The Italian results support the use of palivizumab prophylaxis for otherwise healthy preterm (29–36 wGA) infants aged < 6 months at the beginning of the RSV season.

## Introduction

Respiratory syncytial virus (RSV) is a major pathogen for lower respiratory infections in infants aged < 1 year [1, 2]. It remains the leading cause of hospitalization in previously healthy infants during their first year of life [3, 4]. RSV infection may be so severe as to require hospitalization in 0.5–2.0% of cases [5, 6] and represents the main cause of death in infants with respiratory infections [2–4, 7, 8]. About one-fifth of preterm infants infected with RSV need intensive care during hospitalization [4, 7], a trend that appears to have grown over the past few years [4]. A major risk for severe RSV infection is prematurity [7–9], not only in extreme preterm infants (< 29 wGA) but also in preterm infants 29–36 wGA [10].

The increased vulnerability of preterm infants results from two major causes, the early interruption of pulmonary development and the physiologic immaturity of the immune system [11, 12]. The interruption of lung development before 36 wGA during the first 2 years of life reduces lung compliance, functional residual capacity and expiratory flows with abnormal gas exchange, even in neonates who do not present with any significant respiratory diseases [12, 13]. The ineffective immune response to the viral infection also leads to a harmful excessive inflammatory reaction that contributes to increased severity of the disease and promotes exacerbations [14–17]. Morbidity and mortality associated with the first infection are higher in preterm infants. The infection severity of the associated functional and structural changes at the airway levels explains the more frequent and clinically more relevant respiratory morbidity throughout subsequent years and in this group of patients [16, 18]. Reports in the literature show that risk factors such as low weight at birth, exposure to maternal cigarette smoking during gestation, chronological age at the start of the RSV epidemic season, male sex, absence or short duration of maternal breastfeeding, attendance at daycare, presence of older siblings and birth order increase the risk of infection [19–25], with a consequent increase in hospitalization risk. The only effective pharmacologic prevention for the RSV infection is prophylaxis with palivizumab, a humanized monoclonal antibody that binds the F protein on the virus surface, blocking fusion of the virus membrane with the target cell membrane [21, 26].

Based on the results of the first clinical studies, palivizumab was authorized in June 1998 by the US Food and Drug Administration and then recommended by the American Academy of Pediatrics (AAP), for the prevention of RSV-induced severe distal respiratory tract disease in pediatric patients at the greatest risk of contracting the severe form of the disease, including preterm infants born at ≤35 wGA [27]. In 2014, a new and more restrictive version of the AAP recommendations was published [28]. For previously healthy preterm infants, palivizumab prophylaxis was recommended only in the group of infants born at < 29 wGA (28 weeks ±6 days) and aged < 12 months at the beginning of the RSV season. As a consequence, the Italian Medicines Agency (AIFA) in 2016, adopted the restrictions recommended by the AAP, resulting in palivizumab financial coverage by the National Health System being limited to infants born at < 29 wGA and aged < 12 months at the beginning of the RSV season [29]. However, in November 2017, in consideration of new clinical data, the AIFA extended the prophylaxis reimbursement to preterm infants > 29 wGA and chronological age of < 6 months, at the beginning of the season [30].

In this review, we report the results from six Italian pediatric institutes and from the Italian Network of Pediatric Intensive Care Units (TIPNet)* on the effects related to the limitation of palivizumab reimbursement. We focus on Italian data and hospitalizations of infants aged < 12 months with 29–36 wGA. The data spans from the two seasons before (2014–2015 and 2015–2016) through the season after (2017–2018) the application of the 2016 AIFA recommendation.

* a unique Italian database of 15 Pediatric Intensive Care Units collecting epidemiological data on a voluntary basis.

## Methods

### Search strategy and selection criteria

The articles included in this review were retrieved by searching PubMed for publications on RSV in Italy, dated from 2017 to include the clinical studies from 2016 to 2017; the query is the following:

(rsv [All Fields] OR (“respiratory syncytial viruses”[MeSH Terms] OR (“respiratory”[All Fields] AND “syncytial”[All Fields] AND “viruses”[All Fields]) OR “respiratory syncytial viruses”[All Fields] OR (“respiratory”[All Fields] AND “syncytial”[All Fields] AND “virus”[All Fields]) OR “respiratory syncytial virus”[All Fields]) OR (“bronchiolitis”[MeSH Terms] OR “bronchiolitis”[All Fields]) AND (“palivizumab”[MeSH Terms] OR “palivizumab”[All Fields]) OR (“palivizumab”[MeSH Terms] OR “palivizumab”[All Fields] OR “synagis”[All Fields]) AND (“italy”[MeSH Terms] OR “italy”[All Fields])) AND (“2017”[PDAT]: “3000”[PDAT]).

The query returned 11 articles that had been peer reviewed. Of these, three were retained and eight were excluded (two on pre-clinical aspects, two reviews, two on special populations and two with data from administrative databases). In addition to the three manuscripts by Capizzi et al. [31] and by Picone et al. [32, 33], the review also included abstracts and posters presented in 2017, at the following three Congresses of Italian Pediatric Societies: the Italian Society for Respiratory Diseases in Children, SIMRI (Di Mattia et al.) [34], the Neonatal and Pediatric Society of Anesthesia and Resuscitation, SARNePI (Wolfler et al) [35] and the Italian Society of Neonatology, SIN (Santisi et al. [36], Vittucci et al. [37] and Venafra et al. [38]. Since the data presented on the report from Picone et al. [32] were integrated with the following year data in the second manuscript by Picone et al. [33], we considered this latter report as unique for the purpose of this review. The publications focus on four recent epidemic seasons: two before palivizumab RSV prophylaxis restrictions (2014–2015 and 2015–2016), the season with the palivizumab RSV prophylaxis restriction (2016–2017); and the following one (2017–2018). The data were also compared with the results of an epidemiologic Italian study on RSV-induced lower respiratory tract infection in young children (ALRI) over a four year period (2000–2004), when the use of palivizumab prophylaxis was not widespread in premature infants [39, 40]. In Italy, the RSV winter epidemiologic season generally spans October 1 to March 31 and the RSV diffusion risk generally has a 2-year life cycle (or even 3 years [25]); a peak season outbreak is generally followed by an off-peak season. Whenever possible, we compared data from not only 2 sequential years but also peak seasons versus peak seasons and off-peak seasons versus off-peak seasons. We considered only preterm infants aged < 12 months with 29–36 wGA. The number of patients at the emergency department (ED), number of cases of bronchiolitis, number of infants with RSV, the length of hospital stay, presence of comorbidities and birth order were retrieved by the published data and verified by the authors of the aforementioned publications. Comparison of frequency data for the different seasons was performed with a χ^2^ test or with a Fisher’s exact test. For limited samples we relied on nonparametric tests such as the Wilcoxon-Mann-Whitney test. Two-sided *P* values < 0.05 were considered statistically significant.

## Results

Results under consideration for this review are schematically summarized in Tables 1 and 2.

**Table 1:** Results of the analyzed studies

Author, year [ref]	Study population and evaluations	Years reported	Results
1 | Capizzi, 2017 [31]	Infant hospitalizations for RSV (overall and preterm 29–36 wGA and 33–36 wGA) aged < 12 months	2014–2015, 2015–2016 and 2016–2017	2014–2015 and 2015–2016 Total of 137 and 109 hospitalizations 29–36 wGA = 6.6 and 7.3% RSV 33–36 wGA = 5.1 and 6.4% RSV 2016–2017 Total of 120 hospitalizations 29–36 wGA = 9.2% RSV 33–36 wGA = 8.3% RSV
2 | Picone, 2018 [33]	Preterm infant hospitalizations for bronchiolitis (30–32 wGA) aged < 12 months	2015–2016, 2016–2017 and 2017–2018	2015–2016 and 2017–2018 Bronchiolitis: 6/35 (17%) and 6/56 (10.7%) hospitalizations for bronchiolitis: 3/6 (3/35 pt.: 33%) and 3/6 (3/56 pt.: 5.3%) Age at hospitalizations: 7 and 3 months 2016–2017 Bronchiolitis: 12/47 (26%) Hospitalizations for bronchiolitis: 6/12 (6/47 pt.: 44%) Age at hospitalizations: 4.3 months
3 | Wolfler [35]	Infant hospitalizations for RSV (overall and preterm < 27 wGA, 27–30 wGA and > 30 wGA) aged < 12 months	2014–2015, 2015–2016 and 2016–2017	2014–2015 and 2015–2016 Total hospitalizations for RSV: 88 and 115 < 27 wGA = 0 and 2 (1.7%) 27–30 wGA = 3 (3.4%) and 3 (2.6%) > 30 wGA = 13 (14.8%) and 7 (6.1%) 2016–2017 Total hospitalizations for RSV: 111 < 27 wGA = 0 27–30 wGA = 6 (5.4%) > 30 wGA =27 (24.3%)
4 | Di Mattia [34]	Preterm infant hospitalizations for RSV (< 29 wGA, 30–36 wGA and ≥ 37 wGA) with no age provided	2015–2016 and 2016–2017	2015–2016 < 29 wGA = 0 30–36 wGA = 8 ≥37 wGA = 45 Total of 53 RSV 2016–2017 < 29 wGA = 0 30–36 wGA = 12 ≥37 wGA = 51 Total of 63 RSV *P* = 0.01
5 | Venafra [38]	Preterm infant hospitalizations for RSV (< 35 wGA)	2015–2016 and 2016–2017	2015–2016 RSV: 34/69 (48%) Age at hospitalizations: 3.9 months 2016–2017 RSV: 62/112 (56%) Age at hospitalizations: 3.4 months
6 | Vittucci [37]	Preterm infant hospitalizations for bronchiolitis (< 30 wGA and 30–37 wGA) aged < 12 months	2016–2017	2016–2017 ≤29 wGA, treated = 3/3 30–37 wGA, treated, *n* = 2/29 RSV, treated, ≤37 wGA, n (%) = 1/5 (20%) RSV+, not treated, 30–37 wGA, n (%) = 13/27 (48.2%)
7 | Santisi [36]	Infant hospitalizations for bronchiolitis (overall and preterm < 29 wGA, 29–32 wGA and 32–35 wGA) aged < 6 months and 6–12 months	2016–2017	2016–2017 < 6 months: 55.9% hospitalizations for bronchiolitis (60% RSV) 6–12 months: 25.9% hospitalizations for bronchiolitis (39% RSV) < 29 wGA =1.5% hospitalizations for bronchiolitis 29–32 wGA =2.4% hospitalizations for bronchiolitis 32–35 wGA = 7% hospitalizations for bronchiolitis

*pt* patients, *RSV* respiratory syncytial virus, *wGA* weeks of gestational age. The season of 2016–2017 is the endemic season of the AIFA limited palivizumab prophylaxis prescription coverage

**Table 2:** Summary table of the Italian data by hospital

Season	Hospital	Population, n	Infants with bronchiolitis (30–35 wGA), n (%)	Infants with RSV (30–35 wGA), n (%)	Aged < 6 months, n (%)	Aged < 3 months, n (%)	HFNC, n (%)	Birth order, n (%)
2014–2015	“G. Gaslini” Instit. Genova	137 (RSV induced ALRI)		9 (6.6)	6 (66.7)	5 (55.6)	7 (77.8)	5 (55.6) first born 4 (44.4) not first born
2015–2016	“Umberto I” Hosp. Roma	152 (bronchiolitis)	14 (9.2)*	8 (57.1)				
	“G. Gaslini” Instit. Genova	109 (RSV induced ALRI)		8 (7.3)	3 (37.5)	3 (37.5)	2 (25)	8 (100) not first born
	“Casilino” Hosp. Roma	35 (newborn 30–32 wGE)	6 (17)**				1 (16.7)	
2016–2017	“Umberto I” Hosp. Roma	132 (bronchiolitis)	18 (13.6)*	12 (66.7)				
	“G. Gaslini” Instit. Genova	120 (RSV induced ALRI)		11 (9.2)	9 (81.8)	9 (81.8)	9 (81.8)	2 (18.2) first born 9 (81.8) not first born
	“Casilino” Hosp. Roma	47 (newborn 30–32 wGE)	12 (25.5)**				2 (16.7)	
2017–2018	“Casilino” Hosp. Roma	56 (newborn 30–32 wGE)	6 (10.7)				1 (16.7)	

HFNC = High Flow Nasal Canula; RSV = respiratory syncytial virus; wGA = weeks of gestational age. *P = 0.05; **P = 0.184

The study from Capizzi et al. reports the data for infants aged < 1 year who had been hospitalized for RSV-induced respiratory infections at the “Giannina Gaslini” Institute Pediatric Hospital of Genoa [31] for three consecutive epidemic seasons, from 2014 to 2015 through 2016–2017. Data comparison for three consecutive seasons, including the season 2016–2017 with running restrictions, shows a high rate of hospitalizations, mostly related to infants 33- < 36 wGA, a chronological age of < 6 months and at least one risk factor. Furthermore, during the 2016–2017 epidemic season, a larger percentage of preterm infants received high-flow nasal cannula ventilation, especially in the 33- < 36 wGA group [31].

The clinical study conducted at the “Casilino” General Hospital in Roma by Picone et al. [32, 33] retrospectively analyzed data related to 30 (30 + 0)-32 (32 + 6) wGA preterm infants, aged < 12 months in three seasons: 2015–2016, 2016–2017 and 2017–2018. The number of infants who received palivizumab prophylaxis was markedly lower in 2016–2017 (the season with the active AIFA restrictions) compared with 2015–2016 and 2017–2018, during the restriction only 2 children were treated (2/47 or 4%) compared with 27 treated children (27/35 or 77%) the season before restriction and the whole cohort the year after restriction (56/56 or 100%).The reduced palivizumab prophylaxis was associated with a higher incidence of bronchiolitis in 2016–2017 [26% (12/47)] compared with 2015–2016 (17%, 6/35) and 2017–2018 (10.7%, 6/56). Of note, the three infants who received palivizumab prophylaxis and were hospitalized during 2017–2018 had received only one palivizumab dose before contracting the infection.

**Fig. 1:**
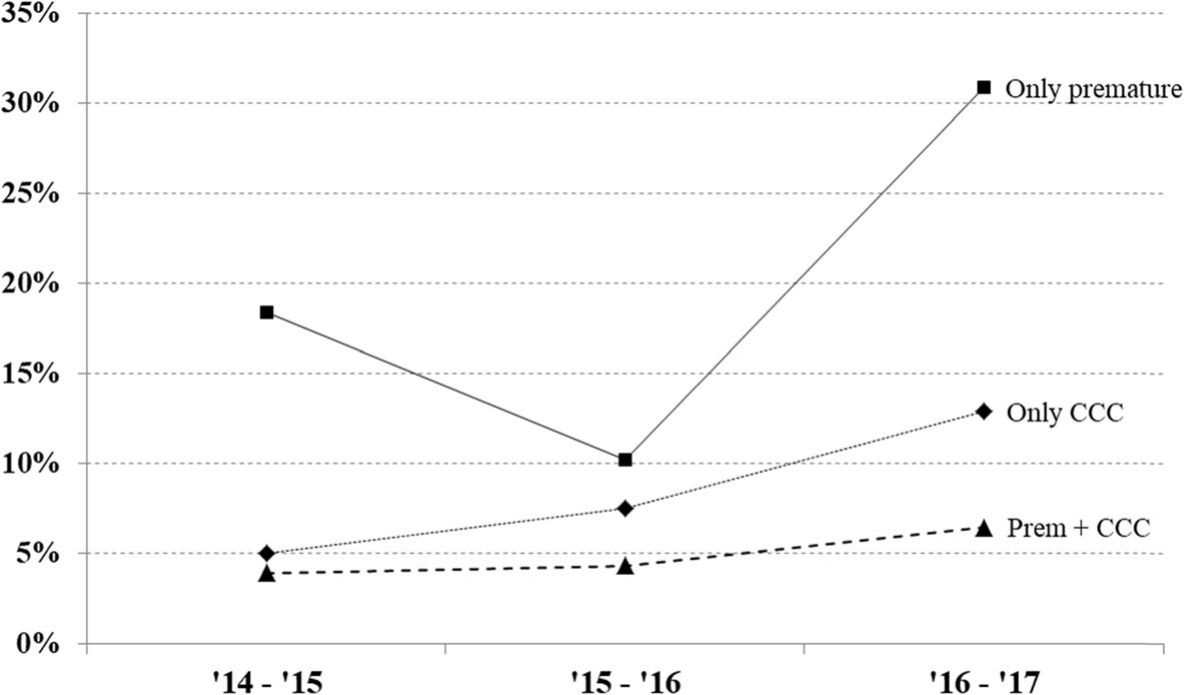
Patient percentage distribution across 3 epidemic seasons for premature with and without comorbidities and not premature infants with comorbidities

**Fig. 2:**
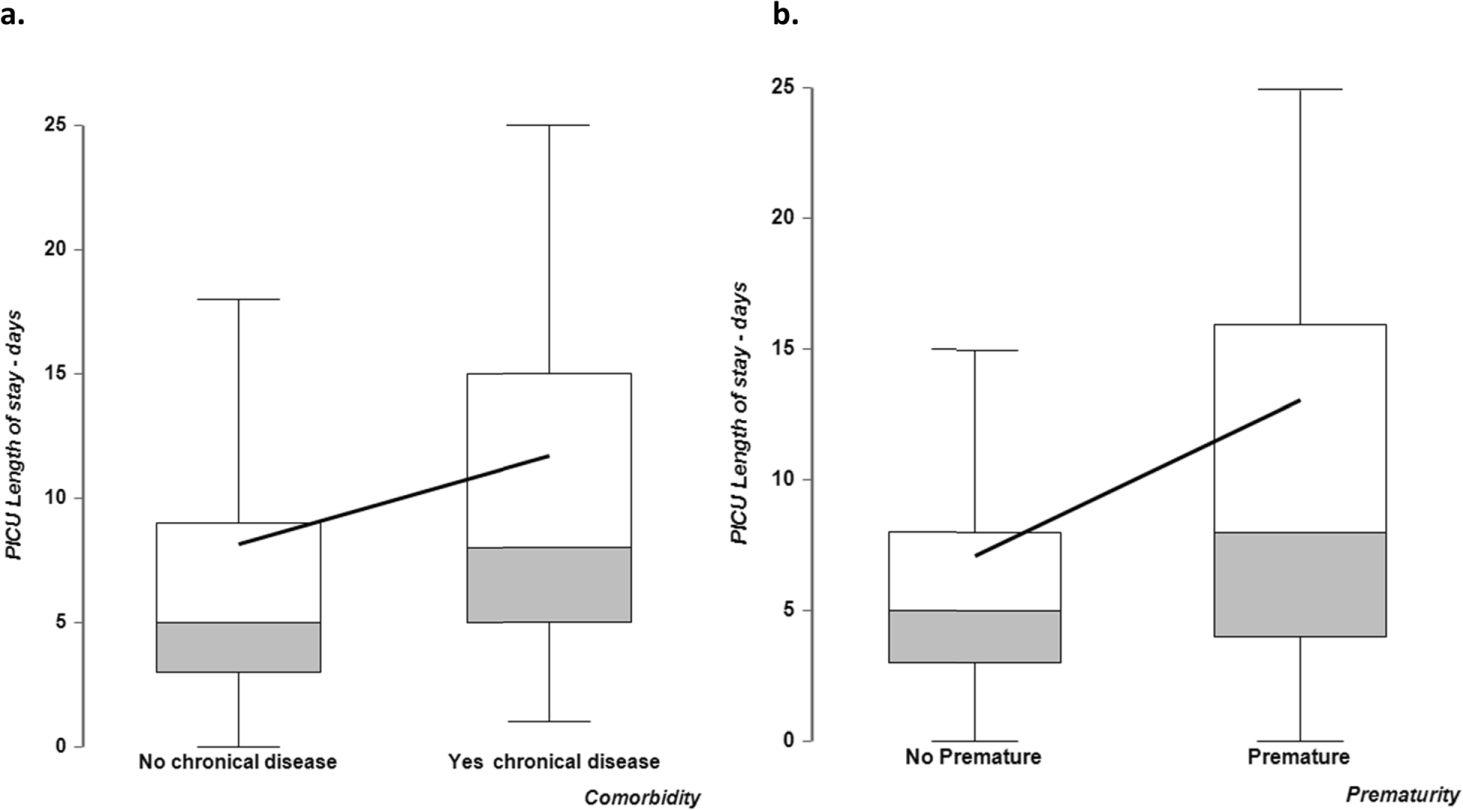
**a**. Length of stay, in days, for infants with and without chronic disease, **b**. Length of stay in days for premature infants versus not premature

The multi-center, retrospective observational study by Wolfler et al. [35], assessed the frequency of admissions for RSV bronchiolitis-induced respiratory insufficiency to the Pediatric Intensive Care Units (PICUs) of TIPNet. Specifically, this study focused on data related to infants aged < 12 months over three consecutive seasons 2014–2015, 2015–2016 and 2016–2017 (Fig. 1). A total of 314 hospitalizations in PICUs were due to RSV bronchiolitis: 88 (27.6%), 115 (36.6%) and 111 (35.4%) in the three epidemic seasons, respectively (Table 3). The comparison between the two peak seasons, 2014–2015 and 2016–2017, shows a statistically relevant increase in the number of infants infected with RSV: 88 in the first one and 111 in the second one (*p* < 0.04). Moreover, there were 13 infants 30–36 wGA hospitalized in the 2014–2015 season, 7 in the 2015–2016 season and 27 in the 2016–2017 season, which had active RSV prophylaxis restrictions (*p* < 0.001) [35]. In the 2016–2017 season, an overall increase of PICU hospitalizations was detected, not only in preterm infants with and without comorbidities but also in full term infants with comorbidities (Fig. 1). As expected, a longer PICU length of stay was also observed for infants with comorbidities, and for preterm babies, as compared respectively with infants without comorbidities, and full-term children (Fig. 2a and b).

**Table 3:** Summary table for the TIPNet data

Season	Population composition (TIPNet), n	Preterm and no comorbidity, n (%)	Full term and comorbidity, n (%)	Preterm and comorbidity, n (%)	Length of stay (mean number of days)			
					Without chronic disease	With chronic disease	Preterm	Full term
2014–2015	88	16 (18)	4 (4.5)	3 (3)	6.2	13	14	5
2015–2016	115	12 (10)	8 (7)	3 (3)				
2016–2017	111	35 (32)	14 (13)	6 (5)				

*ALRI* Acute Lower Respiratory Infections, *RSV* respiratory syncytial virus, *TIPNet* Italian Network of Pediatric Intensive Care Units, *wGA* weeks of gestational age

The retrospective study conducted by Venafra et al. reported the incidence of RSV bronchiolitis hospitalizations at the “Di Venere” Hospital of Bari for preterm infants < 35 wGA, before and after the application of the RSV prophylaxis restrictions [36]. The incidences of hospitalizations in < 35 wGA infants for RSV bronchiolitis was 48% (34/69 infants, with average chronological age upon admission of 3.9 months) during the 2015–2016 epidemic season and 56% (62/112 infants, with average chronological age upon admission of 3.4 months) during the “prophylaxis restrictions” 2016–2017 epidemic season.

The study conducted during the 2016–2017 epidemic season at the Pediatric and Infectious Disease Unit of the Pediatric Hospital “Bambino Gesù” of Rome by Vittucci et al. [37] included 194 infants hospitalized for bronchiolitis (105 males), of whom 162 were born full term (group A) and 32 preterm (group B). In group B, three patients with < 30 wGA had received at least one dose of palivizumab, while only two of the 29 patients in the ≥30 to < 37 wGA range had received palivizumab prophylaxis (one with esophageal atresia and one with bronchial dysplasia). Among the group B infants who had received prophylaxis, only one (but that had received a single palivizumab dose) was found to have RSV infection. In contrast, out of the 27 preterm infants who did not receive prophylaxis, 13 (40.6%) were found to have RSV infection. Furthermore, 95 of 194 hospitalized infants (48%) needed oxygen therapy, 77 (81%) in group A and 18 (19%) in group B, and 34 (17%) were treated with continuous positive airway pressure: 23 (68%) in group A and 11 (32%) in group B (*p* < 0.05). Of the 95 patients who needed oxygen therapy, 65 (68%) were found to have RSV infection. Moreover, 17 of 194 patients (8.8%) required admission to the PICU: 10 in group A and 7 in group B, respectively (*p* < 0.05). The mean hospital stay was 4.9 ± 5.5 days for group A and 8.8 ± 4.4 days for group B (*p* = 0.14).

In the same 2016–2017 epidemic season and in the same “Bambino Gesù” pediatric hospital, a second study was conducted at the Pediatric Emergency and PICU Departments by Santisi et al. [36] to measure the incidence of hospitalizations due to bronchiolitis in infants aged < 12 months. Of the 890 infants diagnosed with bronchiolitis at their arrival at the ED, 82% were aged < 6 months. The admission rate was 55.9%, in the < 6 months old group, and 25.9%, in the 6–12 months old group and 1.5, 2.4 and 7%, respectively in the < 29, 29–32 and > 32–35 wGA group. The percentages of infants found to have RSV were 60% in those aged < 6 months and 39% for those aged ≥6 months. The mean hospital stays were 7.2 days (± 5.2 days) in those aged < 6 months and 8.6 days (±14.2 days) in those aged ≥6 months. The ICU rates of admission were 6% for those aged < 6 months and 7.5% in those aged ≥6 months.

**Fig. 3:**
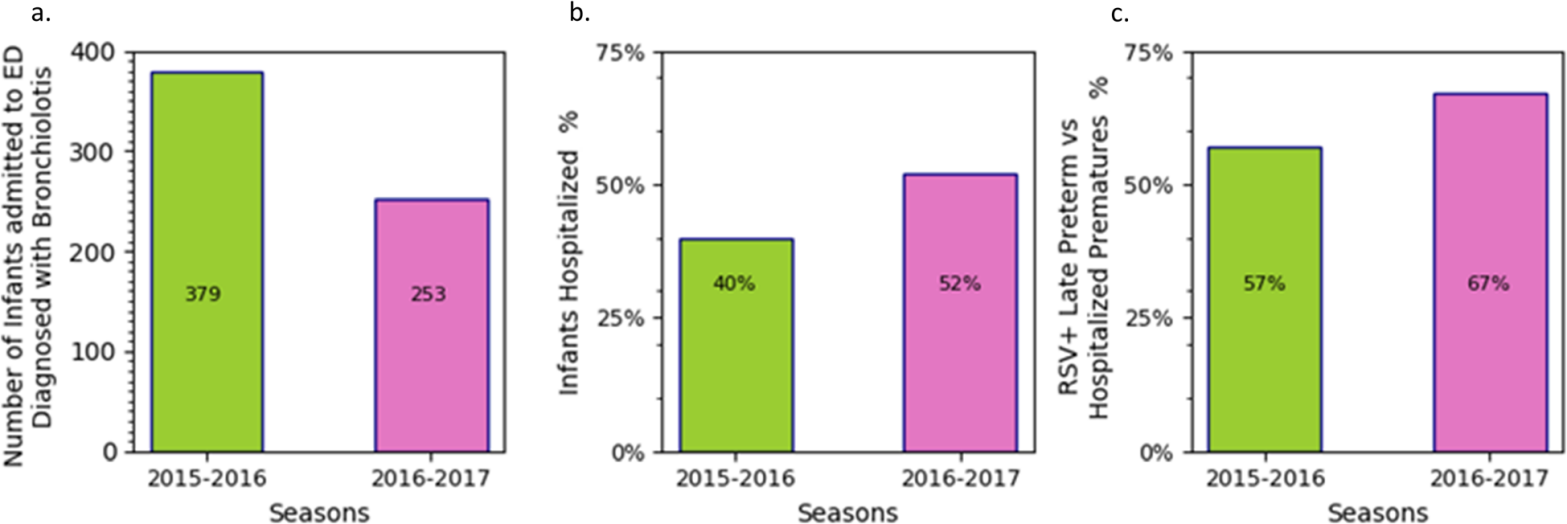
Histograms reporting for two consecutive seasons, 2015–2016 and 2016–2017, on the number of infants admitted to the ED, the infants hospitalized and late preterm infants with RSV. For the 2015–2016 and 2016–2017 seasons, (**a**) the number of infants admitted to the ED who are diagnosed with bronchiolitis, (**b**) the percentages of infants hospitalized for bronchiolitis relative to the number admitted to the ED, and (**c**) the percentages of preterm infants with RSV relative to the number of hospitalizations (379 and 253 as reported in panel **a**) for bronchiolitis in preterm infants. Refer to Table 2 for the absolute values from where such a percentage had been derived

At the Pediatric Department “Umberto I” University Hospital, University of Roma “La Sapienza”, Di Mattia et al. reviewed the clinical charts of infants hospitalized for bronchiolitis during the 2015–2016 and 2016–2017 epidemic seasons [32]. The numbers of infants admitted to the ED with a diagnosis of bronchiolitis in the 2015–2016 and 2016–2017 seasons were 379 and 253, respectively, while the numbers of those hospitalized were 152 (40%) and 132 (52%), respectively (Fig. 3). Selecting only preterm infants 30–36 wGA, 14 were hospitalized (9% with bronchiolitis) in the first season and 18 (14% with bronchiolitis) in the second season. The numbers of hospitalized infants with RSV were 53 (35%) and 63 (48%) in the first and second seasons, respectively (*P* = 0.01), while the numbers of hospitalized infants with RSV with 30–36 wGA were 8 (15%) and 12 (19%) for the 2015–2016 and 2016–2017 seasons, respectively.

## Discussion

During the RSV epidemic season 2016–2017, the 2014 AAP recommendations were applied in Italy, limiting the anti-RSV palivizumab prophylaxis to otherwise healthy preterm infants with < 29 wGA and aged < 12 months at the beginning of the RSV season. However, due to the differences in social and epidemiologic factors, the eligibility criteria to receive palivizumab may differ among countries, particularly for otherwise healthy late preterm infants. In support, we recall that in Italy the only season with palivizumab prophylaxis prescription restrictions was the 2016–2017. Indeed, due to the effect on disease severity and hospitalizations shown by some Italian clinical studies, in November 2017 the new prescription limitations were removed. These initial clinical observations are further strengthened by the comparison of data across endemic seasons discussed in this review.

The results from Capizzi et al. [31] on the RSV-associated hospitalizations for the two seasons before and one season after the AIFA coverage limitation support the need to reassess the role of palivizumab prophylaxis in preterm infants with ≥29 wGA, especially in the presence of specific risk factors. Despite the limitations of this study (data were only collected in a single tertiary level pediatric hospital and on a small number of preterm infants) the results on RSV hospitalization and respiratory support clearly highlight the vulnerability of preterm infants of any gestational age. The AAP 2014 recommendations and the subsequent 2016–2017 AIFA coverage limitations led to an increase in infant exposure to RSV, suggesting a re-evaluation of the recommendation against palivizumab prophylaxis for the subpopulation of infants with > 29 wGA.

The marked reduction in the number of infants who received palivizumab prophylaxis in the restricted season was associated with a higher incidence of bronchiolitis in the publications by Picone et al. [32, 33]. A comparison of the data from the restricted season with that of the seasons before and after showed that palivizumab prophylaxis effectively protected preterm infants from RSV bronchiolitis in nearly all cases, including the most severe forms requiring hospitalization in PICU and intubation. An overall increase of RSV infection in preterm infants with and without comorbidities in the season with limited prescriptions was shown in the multi-center study by Wolfler et al. [35]. The study also showed that hospital stays for infants with comorbidities admitted to the PICU were nearly twice that of infants with no risk factors. A general larger distribution of bronchiolitis and RSV infections among infants with a chronological age < 1 year for the season with running restrictions was shown by Di Mattia et al. [34]. The distribution affected both very preterm infants (< 29 wGA) and a larger number of mid- to late preterm infants (with 29–36 wGA). The tight prophylaxis restriction led to a higher burden of bronchiolitis during the first semester of life and an overall increase in RSV positive bronchiolitis requiring hospitalization during the first year of life. The studies also underscore the respiratory vulnerability of mid- to late preterm infants [34, 35].

In addition to preterm birth, comorbidities and birth order are primary risk factors that also should be systematically considered. Capizzi et al. compared comorbidities and birth order for two peak seasons (2014–2015 versus 2016–2017). The data showed an increase of hospitalizations in non-first-born infants and attendance in a community setting in the restricted season [31]. Results from the Di Mattia et al. report [34] on infant hospitalizations can be compared with those of the Osservatorio study [40]. Of note, the increase in the number of infants hospitalized for RSV in the restricted season in the Di Mattia et al. study was significantly higher compared with infants in the Osservatorio study (*p* = 0.01).

Once an infant has been hospitalized, a relevant parameter to define the severity of the RSV infection is the length of hospital stay. Information about hospital stay is available from Capizzi et al. [29] and the TIPNet database (referred to as intensive treatment) [33]. TIPNet data showed a median hospital stay of 5 days for non-premature infants and 7 days for premature infants, with an upper 95% confidence interval of 15 and 31 days respectively.

A limitation of this systematic review is that, out of the seven reports, five are abstracts presented to congresses, therefore less detailed than peer reviewed manuscripts and reporting only some of all the possible variables that might influence the RSV infection. No questions that comparisons among data reported in full manuscripts would have been preferable.

## Conclusions

Recent Italian RSV data on preterm infants with ≤36 wGA confirm a general high vulnerability for this patient category, specifically showing how the decreased palivizumab prophylaxis in the 2016–2017 epidemic season was associated with a higher incidence of RSV bronchiolitis, a greater respiratory function impairment, and with increased hospitalizations and PICU admission rates. Vulnerability appears to be relevant in infants born with 32- < 36 wGA and the presence of another risk factor. The detection of an overall increase of RSV bronchiolitis cases during the restricted 2016-2017 epidemic season, even in infants born full term, suggests that the decreased palivizumab prophylaxis in preterm infants may have caused the transmission of the infection to infants who are not considered to be at risk.

## Data Availability

The data that support the findings of this study are available within the article and via the referenced articles (depending on institution agreement referenced article might not be free of charge or open-access).

## Abbreviations

AAP: American Academy of Pediatrics
AIFA: Italian Medicines Agency
ALRI: Acute Lower Respiratory Infections
ED: Emergency department
ICU: Intensive care unit
PICU: Pediatric intensive care unit
RSV: Respiratory syncytial virus
TIPNet: Italian Network of Pediatric Intensive Care Units
wGA: Weeks of gestational age
